# A High-Sensitivity, Low-Noise, and Low-Hysteresis Tunneling Magnetoresistance Sensor Based on Structural Optimization of Magnetic Tunnel Junctions

**DOI:** 10.3390/s25061730

**Published:** 2025-03-11

**Authors:** Ran Bi, Ruiying Chen, Shilin Wu, Haoyu Ma, Huiquan Zhang, Xinting Liu, Jinliang He, Jun Hu

**Affiliations:** Department of Electrical Engineering, Tsinghua University, Beijing 100084, China; br20@mails.tsinghua.edu.cn (R.B.); chenry@mail.tsinghua.edu.cn (R.C.); wushilin@tsinghua.edu.cn (S.W.); mahy19@mails.tsinghua.edu.cn (H.M.); zhq22@mails.tsinghua.edu.cn (H.Z.); xt-liu22@mails.tsinghua.edu.cn (X.L.); hejl@tsinghua.edu.cn (J.H.)

**Keywords:** tunnel magnetoresistance, magnetoresistance sensor, annealing process, structural optimization, magnetic hysteresis

## Abstract

Accurate measurement of magnetic fields holds immense significance across various disciplines, such as IC circuit measurement, geological exploration, and aerospace. The sensitivity and noise parameters of magnetic field sensors play a vital role in detecting minute fluctuations in magnetic fields. However, the current detection capability of tunneling magnetoresistance (TMR) is insufficient to meet the requirements for weak magnetic field measurement. This study investigates the impact of structural and fabrication parameters on the performance of TMR sensors. We fabricated series-connected TMR sensors with varying long-axis lengths of the elliptical cross-section and adjusted their performance by modifying annealing magnetic fields and magnetic field bias along the easy axis. The results demonstrate that TMR sensitivity decreases with increasing long-axis length, increases initially and then decreases with an annealing magnetic field, and decreases with a higher bias magnetic field along the easy axis. The voltage noise level of TMR sensors decreases as the long-axis length increases. Notably, the detection capability of TMR sensors exhibits a non-monotonic dependence on long-axis length. Moreover, we optimized the hysteresis of TMR sensors by applying a magnetic field bias along the easy axis. When the bias along the easy axis reached 16 Oe or −40 Oe, the hysteresis level was reduced to below 0.5 Oe. After encapsulating the TMR devices into a full Wheatstone bridge structure, we achieved a detection capability of 17 nT/$\sqrt{\mathrm{Hz}}$@1Hz. This study highlights that the detection capability of TMR devices is jointly influenced by fabrication parameters. By optimizing parameter configuration, this work provides theoretical guidance for further enhancing the performance of TMR devices in magnetic field measurements.

## 1. Introduction

The measurement of weak magnetic fields is of great significance in various areas [1,2,3], including IC circuit testing, geological exploration, and aerospace applications. To meet the requirement of precise detection, various technologies have been developed. Among these, magnetoresistance sensors have emerged as a promising approach due to their potential for high sensitivity, compact size, and low-cost implementation [4]. In recent years, these sensors have garnered widespread research interest in various areas, such as biotechnology [5], the automotive industry [6], geomagnetic detection [7], and deep-space and deep-sea exploration [8]. Tunneling magnetoresistance (TMR) is a kind of magnetoresistance device characterized by high sensitivity and capability of achieving a substantial magnetoresistance ratio (MR) at room temperature. Specifically, MgO-based barrier layers contribute to the high MR and sensitivity [9].

TMR has a wide range of applications. In medium to large magnetic field measurements, it can be utilized in scenarios such as current measurement in power systems and vehicle energy management systems [6,10]. These applications place high demands on the accuracy and measurement range of TMR sensors. In contrast, applications that involve weak magnetic field measurements, such as IC testing [11], biomedical sensing [5], and evaluation of the current state of leakage current in power systems [12,13,14], require TMR sensors with detection capabilities at the nT or even pT level. However, the current detection capabilities of TMR sensors fall far short of meeting the requirements for weak magnetic field measurement.

Current research typically optimizes the performance of small magnetic field detection through three main approaches: regulating the intrinsic parameters of TMR sensors, utilizing on-chip magnetic flux concentrators (MFC), and employing signal modulation techniques [15]. The use of MFC to enhance the sensitivity of TMR sensors fundamentally amplifies the magnetic field, thereby achieving higher equivalent sensitivity. For example, J. Valadeiro et al. [16] achieved a 90-times sensitivity gain using a dual-layer MFC structure, while Marília Silva et al. [17] obtained a 436-times sensitivity enhancement through an optimized MFC design.

Another critical parameter affecting the detection capability of TMR sensors is noise, which consists of shot noise, thermal noise, and 1/*f* noise [18,19,20], with 1/*f* noise dominating at low frequencies. Researchers have adopted signal modulation techniques to reduce or shift low-frequency noise, thereby avoiding high noise at low frequencies. For example. Tomoya Nakatani et al. [21] applied an alternating magnetic field through printed coils to achieve noise modulation, while Du Qingfa et al. [22] employed a “zero-magnetic” modulation strategy, utilizing the periodic movement of a modulation film to compensate for TMR noise.

However, both optimization methods focus on the parameters of magnetic tunnel junction (MTJ) and apply optimization measures externally, while ignoring the regulation of TMR intrinsic performance. The performance of TMR is highly correlated with the structure, size, and other parameters of the thin film. Optimizing the intrinsic parameters of MTJ itself is an effective strategy to improve TMR performance.

Various methods have been employed to improve the sensitivity and noise parameters during the optimization of TMR structural and process parameters. For instance, sensitivity can be improved by incorporating half-metallic materials such as Heusler alloys as layers or by modifying the parameters of the MTJ films. Yujie Hu et al. demonstrated through simulations that the high spin polarization rate of Heusler alloys significantly improves the sensitivity of TMR devices [23]. However, the lattice mismatch between the Heusler alloy and MgO still requires further optimization. The high temperature coefficient of Heusler alloy also hinders its further application [24,25]. In addition, Takafumi Nakano’s team explored the impact of free layer thickness on sensitivity, showing that sensitivity increases with the decrease in thickness. However, when the free layer thickness falls below 1.65 nm, the vertical magnetic moment component restricts further sensitivity improvements. This phenomenon indicates that it is difficult to achieve additional sensitivity enhancement simply by adjusting the thickness of the TMR thin films [26]. Rasly’s team used a two-step annealing process, maintaining the high performance of the TMR [27].

Thus, current methods for improving TMR sensitivity face limitations, and no approach has yet achieved a significant increase in sensitivity.

Zhenhu Jin et al. proposed a simple and feasible strategy by adjusting the dimensional parameters of a rectangular cross-section to modulate the shape anisotropy field ($H_{k}$), thereby controlling both sensitivity and noise [28,29]. However, research suggests that the easy axis of the free layer tends to align parallel to the long axis of elliptical cross-section [30], making elliptical cross sections more suitable for optimizing the TMR performance parameters. The use of elliptical shapes as the planar structure of TMR devices is, therefore, more conducive to achieving optimized performance.

Modifying the shape parameters of TMR devices fundamentally adjusts their anisotropy field strength. In addition to shape parameters, factors such as magnetization bias and magnetic fields applied during annealing also influence the anisotropy field along the TMR easy axis ($H_{\mathrm{AF}}$). A comprehensive adjustment of these parameters provides a straightforward approach to enhancing the sensitivity of TMR devices. TMR devices with different geometric parameters may exhibit different noise characteristics resulting in different detection capabilities. Additionally, hysteresis is another critical parameter for TMR applications. Variations in fabrication parameters can result in differing levels of hysteresis, thereby influencing device performance. For example, Huang et al. [31] optimized the hysteresis level of TMR devices using an AC modulation approach.

To improve TMR detection capability, a combination of simulations and experiments was employed. First, a basic model of the TMR parameters was established for simulation. Elliptical cross-sections sensors with different long-axis lengths were fabricated. During annealing, various magnetic field conditions were applied. Different bias fields were applied to the sensors to get different working conditions. The study systematically examined the resistance of TMR wafer, output voltage of the TMR Wheatstone bridge structure, and noise and hysteresis response, verifying the feasibility of the fabrication approach and identifying the optimal combination of fabrication parameters to achieve the best detection capability.

## 2. Theoretical Analysis

### 2.1. Analysis of Sensitivity and Noise in TMR

In the area of small magnetic field measurements, the core parameters of TMR sensors are sensitivity (*S*) and noise ($S_{v}$), both of which determine the detection capability of TMR.

**Figure 1:**
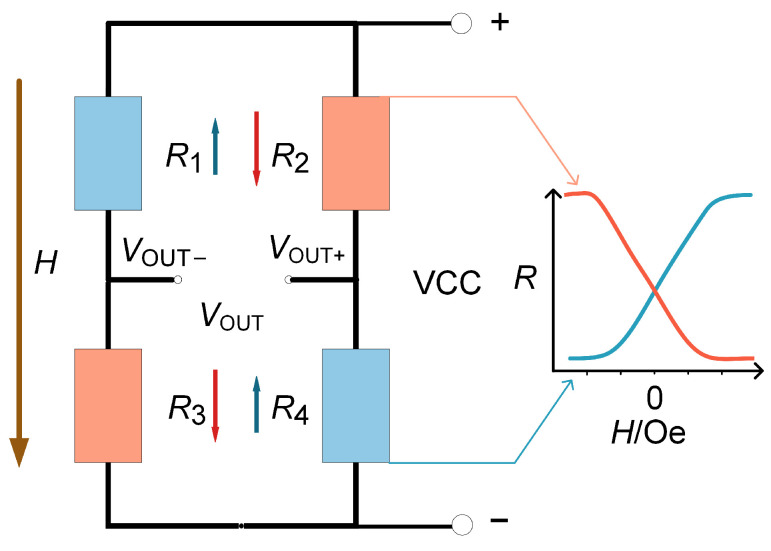
The Wheatstone bridge structure of the TMR, where $R_{1}$ and $R_{4}$ are considered identical, $R_{2}$ and $R_{3}$ are identical, the sensitive axes of the TMR in adjacent arms are oriented in opposite directions, and *H* is the external magnetic field.

For an individual TMR, sensitivity can be directly expressed as *S* = Δ*R*/Δ*H*, where Δ*R* and Δ*H* are the variations in magnetoresistance and magnetic field, respectively. If we consider the Wheatstone bridge structure shown in Figure 1 as the output of the TMR, the output results are as shown as (1).

(1) $$V_{\mathrm{OUT}}=V_{\mathrm{OUT}+}-V_{\mathrm{OUT}-}=\left(\frac{R_{4}}{R_{2}+R_{4}}-\frac{R_{3}}{R_{1}+R_{3}}\right)\times \mathrm{VCC}$$

where *VCC* is the supply voltage, ${R\prime }_{x}$ represents the resistance in Figure 1 when $V_{\mathrm{OUT}}$ is at its maximum, and ${R\prime \prime }_{x}$ represents the resistance when $V_{\mathrm{OUT}}$ is at its minimum.

Assume that the resistance of the TMR varies linearly and has a continuously near-zero magnetic field. Besides, under an applied magnetic field, the increase and decrease in resistance of adjacent arms of the Wheatstone bridge structure are equal. (1) can reach the sensitivity of the TMR sensors expressed as (2).

(2) $$S=\left[\left(\frac{\Delta R_{H}}{2R_{\mathrm{ave}}}-\frac{-\Delta R_{H}}{2R_{\mathrm{ave}}}\right)\right]\times \frac{VCC}{\Delta H_{L}}=\frac{\Delta R_{H}}{R_{\mathrm{ave}}}\times \frac{VCC}{\Delta H_{L}}$$

where $\Delta R_{H}$ represents the change in resistance within the linear magnetic field range $\Delta H_{L}$ and $R_{\mathrm{ave}}$ is the average resistance between the maximum and minimum resistances in the linear range of the TMR.

According to (2), the sensitivity of the TMR sensor is determined by the linear magnetic field range and the ratio of resistance change. Sensitivity can be enhanced by increasing the ratio of resistance change and reducing the magnetic field range.

The noise level of TMR, $S_{v}$, also determines the measurement capability of the magnetic field sensor. It mainly consists of thermal noise ($S_{\mathrm{therm}}$), shot noise ($S_{\mathrm{shot}}$), electrical noise ($S_{\mathrm{elect}-1/f}^{\mathrm{MTJ}}$), and magnetic noise ($S_{\mathrm{mag}-1/f}^{\mathrm{MTJ}}$), and can be expressed as (3).

(3) $$S_{v}=S_{\mathrm{therm}}+S_{\mathrm{shot}}+S_{\mathrm{elect}-1/f}^{\mathrm{MTJ}}+S_{\mathrm{mag}-1/f}^{\mathrm{MTJ}}$$

Among these noises, thermal noise is the intrinsic noise of electronic devices and is difficult to reduce, usually serving as the fundamental limiting factor of the device. Electrical 1/*f* noise and magnetic 1/*f* noise are frequency-dependent and are the primary limiting factors in low-frequency signal measurements. Based on the empirical Hooge constant [9,18,32], the frequency-dependent components of electrical noise and magnetic noise can be expressed as (4).

(4) $$\begin{array}{ccc} \; & S_{\mathrm{elect}-1/f}^{\mathrm{MTJ}}=\frac{\alpha_{\mathrm{elect}-1/f}I_{\mathrm{MTJ}}^{2}R_{\mathrm{MTJ}}^{2}}{Af^{\beta }}\; & S_{\mathrm{mag}-1/f}^{\mathrm{MTJ}}=\frac{\alpha_{\mathrm{mag}-1/f}B_{\mathrm{SF}}}{\Omega f} \end{array}$$

where $\alpha_{\mathrm{elect}-1/f}$ is the Hooge constants for the electrical and $\alpha_{\mathrm{mag}-1/f}$ is the normalized 1/*f* magnetic noise parameter (Hooge-like). $I_{\mathrm{MTJ}}$ is the current flowing through the TMR, $R_{\mathrm{MTJ}}$ the resistance of the TMR, *A* is the MTJ area of the TMR, and *f* is the frequency of the signal being measured. $B_{\mathrm{SF}}$ is the saturation field of free layer. Ω is the volume of free layer. This formula indicates that there exist noises in TMR that are similar to the noise in electronic conduction systems and can be characterized by a Hooge-like parameter.

However, considering noise and sensitivity as two independent parameters is insufficient to directly assess the measurement capability of the Wheatstone-bridge-structured TMR. The detection ($D_{V}$) of the TMR is defined as (5).

(5) $$D_{V}=\frac{1}{S}\times \frac{\sqrt{S_{v}}}{\mathrm{VCC}}$$

In applications involving weak magnetic field measurements, higher sensitivity is desired, while low noise is also crucial. Therefore, designing device parameters and obtaining optimized device parameters are necessary in the preparation of TMR sensors.

### 2.2. Sensitivity Analysis Based on the Stoner–Wohlfarth Model

**Figure 2:**
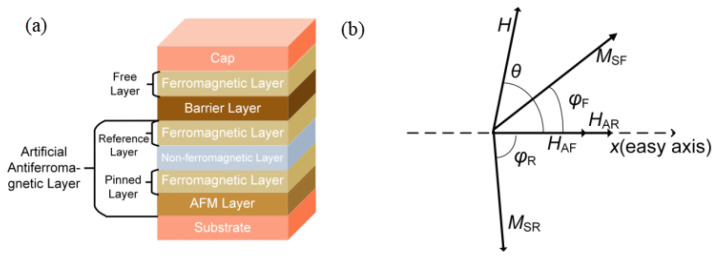
(**a**) Core parameters of TMR, (**b**) Single-domain model of TMR, based on the S-W model considering only the energy of the free layer.

The core structure of the linear TMR sensor consists of an antiferromagnetic layer/ferromagnetic layer/non-magnetic metal layer/ferromagnetic layer/barrier layer/ ferromagnetic layer, as shown in Figure 2a. The magnetic moments of the two ferromagnetic layers adjacent to the antiferromagnetic (AFM) layer are fixed in the hard axis direction under the action of the exchange bias field. The magnetic moment of the free layer is free to rotate with the applied magnetic field.

The angular difference between the directions of the magnetic moments of the free layer ($\varphi_{F}$) and the reference layer ($\varphi_{R}$) determines the magnitude of the output magnetic resistance. The MTJ outputs a minimum resistance ($R_{\min}$) when $\varphi_{F}-\varphi_{R}$ = 0, while the MTJ outputs a maximum resistance ($R_{\max}$) when $\left|\varphi_{F}-\varphi_{R}\right|=180^{\circ }$. The relationship between the output resistance of the linear TMR and the angle between the magnetic moment directions of the reference and free layers is *R* = *R*_*ave*_(1−0.5Δ*R*_*max*_cos($\varphi_{F}-\varphi_{R}$)). $\Delta R_{\max}=\left(R_{\max}-R_{\min}\right)/R_{\mathrm{ave}}$ is the resistance that varies over the range of the magnetic field.

The Stoner–Wohlfarth (S-W) model considers the magnetization reversal behavior of single-domain magnetic particles under an external magnetic field. For TMR sensors, the magnetization of its ferromagnetic layer can be effectively described by the S-W model [33,34,35]. The energy of the S-W model usually considers the free layer, reference layer, and pinned layer. Due to the influence of AFM exchange bias field, the energy of the reference layer and the pinned layer remains almost unchanged under a small magnetic field. Considering only the energy variation in the free layer, a simplified S-W model can be obtained as shown in Figure 2b, with the coordinate x-axis coinciding with the easy axis of the TMR.

Variable $\varphi_{F}$ can be solved according to the steady state energy minimum principle with S-W model. The energy of the free layer ($E_{F}$) primarily consists of Zeeman energy, magnetocrystalline anisotropy energy, and shape anisotropy energy, as expressed in (6).

(6) $$E_{F}=-\mu_{0}M_{\mathrm{SF}}H\cos\left(\varphi_{F}-\theta \right)+\frac{1}{2}\mu_{0}M_{\mathrm{SF}}H_{\mathrm{AF}}\sin^{2}\varphi_{F}$$

where $H_{\mathrm{AF}}$ and $H_{\mathrm{AR}}$ are the anisotropy field of free layer and reference layer, respectively, (θ) is the direction of the magnetic field, *H* is the magnetic field value, and $M_{\mathrm{SF}}$ and $M_{\mathrm{SR}}$ are the magnetic moment of the free layer and reference layer, respectively.

When $M_{\mathrm{SF}}$ reaches a steady state, the magnetization energy of the free layer is minimized. Specifically, when θ is fixed, the variable $\varphi_{F}$ is determined by *H*, satisfying the equality relationship in (7).

(7) $$\begin{array}{cc} \; & \frac{\partial E_{F}}{\partial \varphi_{F}}=\mu_{0}M_{\mathrm{SF}}H\sin\left(\varphi_{F}-\theta \right)+\mu_{0}M_{\mathrm{SF}}H_{\mathrm{AF}}\sin\varphi_{F}\cos\varphi_{F}=0\\ \; & \frac{\partial^{2}E_{F}}{\partial \varphi_{F}^{2}}=\mu_{0}M_{\mathrm{SF}}H\cos\left(\varphi_{F}-\theta \right)+\mu_{0}M_{\mathrm{SF}}H_{\mathrm{AF}}\cos2\varphi_{F}>0 \end{array}$$

Since (7) is a transcendental equation without an analytical solution, numerical methods are required to determine $\varphi_{F}$ for varying values of *H*.

### 2.3. Results of Sensitivity Simulation Based on the S-W Model

Consider three structural parameters of the TMR: the long-axis lengths of the TMR, the annealing parameters, and the magnetic field bias along the easy axis ($H_{\mathrm{BFX}}$). The first two parameters are implicit, and their effects on the sensitivity of the TMR cannot be directly analyzed from the S-W model. However, these can be incorporated into the $H_{\mathrm{BFX}}$, allowing the overall impact of the bias to be analyzed using the S-W model.

Assuming that the TMR constituting each bridge arm have good symmetry, the response of the resistance to the magnetic field is opposite in sign and equal in amplitude in the linear range. The transfer function of the output voltage with respect to the angle of clamping as shown in (8) can be obtained.

(8) $$v_{\mathrm{OUT}}=\frac{\left(\cos\left(\varphi_{F1}-\varphi_{R1}\right)-\cos\left(\varphi_{F2}-\varphi_{R2}\right)\right)}{\left(4-\cos\left(\varphi_{F1}-\varphi_{R1}\right)-\cos\left(\varphi_{F2}-\varphi_{R2}\right)\right)}$$

In this case, $v_{\mathrm{OUT}}$ represents the normalized output voltage relative to the supply voltage. $\varphi_{F1}$ and $\varphi_{R1}$ represent the magnetic moment directions of the free layer and reference layer for the TMR $R_{1}$ and $R_{4}$, while $\varphi_{F2}$ and $\varphi_{R2}$ represent the magnetic moment directions of the free layer and reference layer for the TMR $R_{2}$ and $R_{3}$.

**Figure 3:**
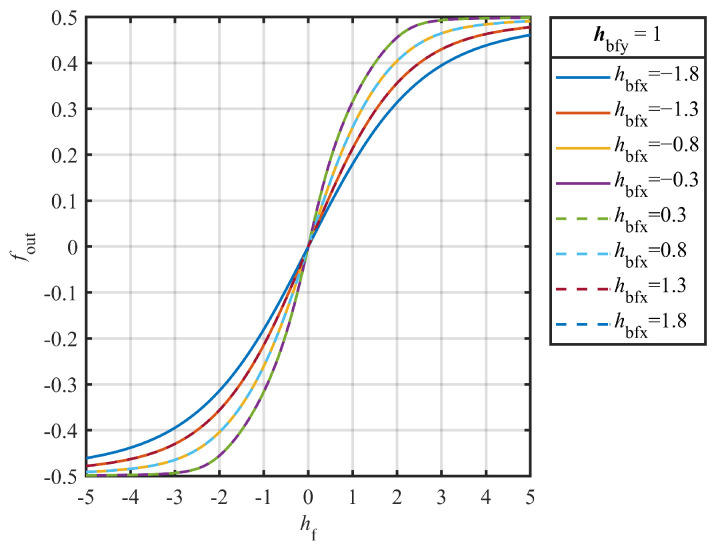
Simulation results of $H_{\mathrm{bfx}}$. The $h_{F}$ on the x-axis is the normalized value of *H* relative to $H_{\mathrm{AF}}$. In the legend, $h_{\mathrm{bfy}}$ and $h_{\mathrm{bfx}}$ represent the normalized value of the bias fields applied along the hard axis and easy axis relative to $H_{\mathrm{AF}}$, respectively. This bias field can be influenced by various factors, such as external bias and the interface parameters. In the simulation, $h_{\mathrm{bfy}}$ was fixed at 1 while changing $h_{\mathrm{bfx}}$ to observe the simulation results, as $h_{\mathrm{bfx}}$ has a greater impact on the sensitivity parameters.

As shown in Figure 3, the normalized value of magnetic field bias along the easy axis $h_{\mathrm{bfx}}$ affects the sensitivity of the TMR. Sensitivity decreases as $h_{\mathrm{bfx}}$ increases, which is reflected by a reduced slope in the graph. Moreover, the symbol $h_{\mathrm{bfx}}$ does not affect the sensitivity. This is represented in the graph by the overlap of the output curve for equal-magnitude positive and negative biases along the easy axis. As the overall bias strength along the easy axis increases, the sensitivity of the TMR decreases. Therefore, to improve TMR sensitivity, the |$h_{\mathrm{bfx}}$| should be minimized during the fabrication process. The artificial AFM structure consists of a multilayered MnIr/CoFe/Ru/CoFe/CoFeB films, where the CoFeB layer serves as the reference layer and is antiferromagnetically coupled with the CoFe layer via the non-metallic Ru layer, fixing its magnetic moment along the hard axis. The barrier layer consists of MgO with a <001> orientation. CoFeB on the other side of the MgO constitutes the free layer, whose magnetic moment direction is free to rotate.

## 3. Experimental Methods

### 3.1. Parameters Design and Fabrication of TMR

**Figure 4:**
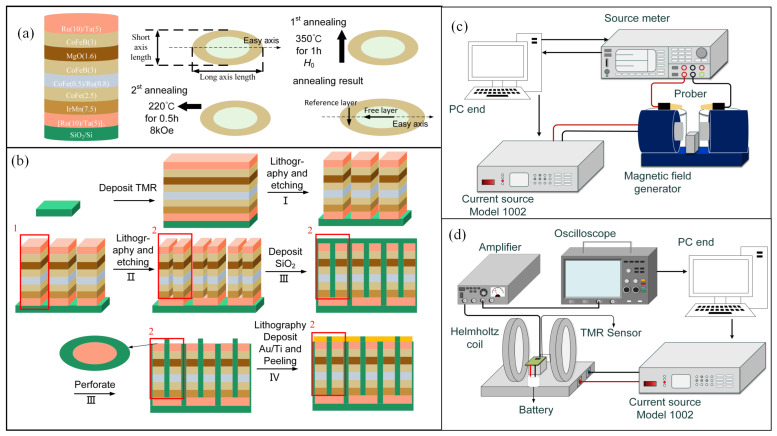
(**a**) The structural parameters of TMR include the thickness of each layer and details about the annealing process, together with the length indication of the elliptical cross-section. (**b**) Fabrication process of the TMR includes a four-step lithography process; the deposition of ${\mathrm{SiO}}_{2}$ and perforate hole creation shares one lithography step, and thus, they are counted as a single step. (**c**) Stepper-based DC magnetic field measurement platform. (**d**) Noise measurement platform for TMR devices.

The MTJ film was deposited using ultrahigh vacuum magnetron sputtering equipment (pressure of less than $3\times 10^{-6}$ Pa). The film structure was Ta(5)/Ru(10)/Ta(5)/Ru(10)/ Ta(5)/Ru(10)/MnIr(7.5)/CoFe(2.5)/Ru(0.8)CoFe(0.5)/CoFeB(3)/MgO(1.6)/CoFeB(3)/Ru(0.8)/ NiFe(3.5)/Ru(10)/Ta(5)/Ru(5) (in nm), as shown in Figure 4a. MnIr is used as the AFM material, fixing the magnetic moment direction of the CoFe layer (the pinned layer) along the hard axis through the exchange bias field. Using <001> oriented MgO as the barrier layer; on the other side of the MgO, CoFeB constitutes the free layer, in which the magnetic moment direction can rotate freely.

The prepared MTJ films were patterned using lithography and etching techniques to create MTJ devices with different long-axis lengths and varying numbers of MTJ in series. The designed TMR have a fixed short-axis length of 4 μm, and five different long-axis lengths were designed to verify the effects of different long-axis lengths on the performance of the TMR sensors.

The primary process is depicted in Figure 4b and involves four lithographic steps. In the step I, the MTJ thin layer was lithographically patterned and etched to form the bottom electrode pattern. The bottom electrode comprised a buffer layer and an artificial AFM structure, as illustrated in Figure 4b red block 1. Subsequently, lithography and etching were performed on the bottom electrode to obtain the elliptical MTJ pattern, including the free layer and the barrier layer, as shown in Figure 4b red block 2. The structure within the red block 2 corresponded to the elliptical shape illustrated in Figure 4a, simplified as a rectangle for convenience in the figure. Serial MTJ devices are interconnected via the bottom electrode, with each bottom electrode linked to four MTJs, indicating a parallel quantity of two. In the step III, an insulating layer was deposited to prevent short circuiting of the top metal of the serial tunnel junctions. Subsequently, photolithography and via-etching techniques are employed to expose the top of the serial devices. In the step IV, deposition of Ti/Au was followed by the lift-off of excess metal.

After completing the fabrication of the serial tunnel junctions, a two-step annealing process is performed on the TMR in a high-vacuum magnetic field annealing furnace, as shown in Figure 4a. At the same time, we assume that the long axis of the TMR elliptical cross-section coincides with the easy axis. This process induces the initial magnetization directions of the TMR reference layer and the free layer to be perpendicular, achieving a monotonic linear interval. A magnetic field is applied along the hard axis and the easy axis during the two-step annealing processes, respectively, to induce the magnetic moment direction of the artificial AFM coupling and the anisotropic field direction of the free layer. Specifically, the first step involves annealing at 350 °C with a magnetic field $H_{0}$ along the hard axis, where $H_{0}$ is varied at 2 kOe, 5 kOe, 6.5 kOe, and 8 kOe. The second annealing step is carried out at 220 °C with an 8 kOe magnetic field along the easy axis.

### 3.2. TMR Measurement Platform and Testing Process

A TMR measurement platform was established, as shown in Figure 4c,d. Figure 4c displays a stepper-based DC magnetic field measurement platform, specifically designed for testing TMR wafers. The resistance variation of the TMR with respect to the magnetic field is measured with a DC probe. The DC current source Model 1002 (magnetoelectricity Technology Co., Ltd, Beijing, China) operates in conjunction with an electromagnet to generate a stepwise varying DC magnetic field. The current stepping precision of the DC current source being 0.1 mA, together with the minimum step size of the DC magnetic field reaching 0.05 Oe. A source-measure unit (Agilent 2912B model, Keysight Technologies, Sunnyvale, CA, USA) is utilized as the resistance measurement device. The source-measure unit is connected to the probe and measures the TMR resistance by applying a voltage and measuring the resulting current. An automated TMR measurement platform was developed on a PC using the LabVIEW 2019 environment. The PC sends control signals to adjust the output current of the DC current source and interacts with the source-measure unit to obtain resistance measurements at various magnetic fields.

Figure 4d illustrates the setup of a noise energy measurement platform. A Mitsubishi Q6BAT battery (Toshiba Battery Co. Ltd., Fukushima city, Japan) is used as a low-noise power source to supply the Wheatstone bridge structure of the TMR sensor. The low-noise differential amplifier (NF5307) is employed to amplify the differential voltage output from the Wheatstone bridge structure. This amplifier has an input noise as low as 4 nV/$\sqrt{\mathrm{Hz}}$@1Hz, allowing it to amplify the differential voltage of the Wheatstone bridge structure without interfering with the TMR noise. The TMR sensor is placed inside a shielding box made of Permalloy to reduce external interference. A high-precision data acquisition card is used to convert the analog signal into a digital signal. The noise power spectral density is obtained through Fourier transformation, allowing for the calculation of the voltage noise level in the Wheatstone bridge structure.

## 4. Result and Discussion

### 4.1. Experimental Results of TMR Wafer Resistance Sensitivity

Measure the magnetic field response of a TMR wafer resistance. A magnetic field was applied to the TMR in a magnetically shielded chamber to measure the response of the TMR to changes in magnetic field. The magnetic field generated by the electromagnet was calibrated by a Gaussmeter (LakeShore Model 425) to obtain a highly accurate magnetic field–current relationship. The current flowing through the TMR wafer was measured by applying a 1 V voltage to both ends of the TMR wafer through the source meter (Agilent 2912B model) to obtain the resistance value of the TMR wafer at different magnetic fields to obtain the sensitivity S of the TMR wafer.

The MR performance of different number of series connections is measured at room temperature, and the annealing magnetic field is 6.5 kOe for TMR device. As an example, the MR at different numbers of series connections is shown in Table 1. TMR with a 64 μm long-axis length cannot achieve more than 128 series connections in a limited area (1200 × 600 μm^2^). Therefore, when the number of series connections exceeds 128, the number of MTJ in 64 μm long-axis length TMR is 192 (two resistors per cell) and 384 (one resistor per cell).

After deposition of the TMR thin layer, the MR was measured directly using a probe and was up to 120.2%. When the number of series connections is less than 64, the resistance of the electrodes used for series connections accounts for a large proportion of the TMR, resulting in an MR much less than the nominal value of 120%. When the number of series connections is greater than 128, the MR increases slightly with the number of series connections and stabilizes above 60%. The number of series connections affects the grain size, which affects the total size of the TMR sensor. Considering that the MR tends to stabilize for TMR series number of 128 and above, 128 or 256 series number was chosen as the design series number for TMR to achieve a larger TMR ratio while the total sensor size is smaller.

**Figure 5:**
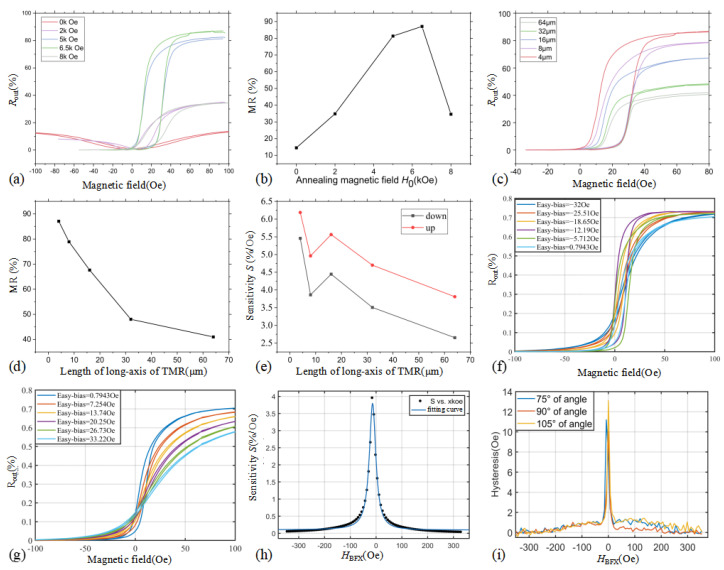
(**a**) Response of TMR resistance to $H_{0}$ with a long-axis length of 16 μm. (**b**) Maximum MR of TMR in as a function of $H_{0}$. (**c**) Response of TMR resistance to the long-axis length. (**d**) Maximum MR of the TMR as a function of the long-axis length. (**e**) Fitting sensitivity curve of the TMR as a function of the long-axis length. (**f**,**g**) TMR output curve with different $H_{\mathrm{BFX}}$. (**h**) Fitting curve of TMR sensitivity and $H_{\mathrm{BFX}}$. (**i**) Hysteresis output curve with $H_{\mathrm{BFX}}$.

TMR devices were prepared under different annealing parameters and the measured results are shown in Figure 5a,b. The curve labeled “0 kOe” represents the unannealed test results, while the other curve corresponds to the results after two annealing steps with different $H_{0}$.

The variation of $H_{0}$ (Figure 5b) shows that the process is unable to fix the direction of the magnetic moment of the reference layer completely on the hard axis when the $H_{0}$ is low (e.g., 2 kOe). On the other hand, if the $H_{0}$ is too high (e.g., 8 kOe), the MR deteriorates. There exists an optimal value of the $H_{0}$ to maximize the MR. Specifically, in this study, the MR reaches its highest value with $H_{0}$ of 6.5 kOe, at which time the sensitivity is maximized.

The effect of long-axis length on the TMR as well as the sensitivity of the TMR wafer is tested for the TMR device parameters with 128 series number, as shown in Figure 5c. The MR of the TMR decreases with the increase of the long-axis length, and Figure 5d illustrates that the MR of the TMR decreases nearly linearly with the increase of the long-axis length. Linear fitting of the rising and falling segments of the TMR is performed separately, and the results are shown in Figure 5e. When the long-axis length of the TMR device increases, the sensitivity *S* decreases. At a long-axis length of 8 μm, a decrease in the slope of the *S* is observed from the fitted curve in Figure 5e. At the same time, the increase in the long-axis length will reduce the hysteresis of the TMR (in Figure 5c), which is about 20 Oe when the long-axis length is 4 μm and about 10 Oe when the long-axis length reaches 64 μm. However, the hysteresis cannot be reduced to 0 by changing the long-axis length of the TMR, and therefore, other options need to be considered to regulate the hysteresis.

The $H_{\mathrm{BFX}}$ will also affect the performance of the sensitivity of the TMR, and the test to analyze the effect of the $H_{\mathrm{BFX}}$ on the sensitivity level is shown in Figure 5f,g. When $H_{\mathrm{BFX}}$ is different, the output sensitivity of the TMR with the magnetic field in the direction of the difficult axis will change, and it reaches the maximum value when the bias is at the level of −12.19 Oe. Analytical fitting of the change in sensitivity yields the results shown in Figure 5h. That is, there exists an optimal result for the sensitivity of the TMR with the bias, and the fitting equation is shown in (9).

(9) $$\begin{array}{c} S=\frac{b_{1}H_{BFX}+b_{0}}{H_{BFX}^{2}+a_{1}H_{BFX}+a_{0}}+c \end{array}$$

where the fitting equation has a goodness-of-fit of 0.9916, and the parameters are *a*_1_ = 26.68, *a*_0_ = 410.2, *b*_1_ = −1.336, *b*_0_ = 842.5, and *c* = 0.09661. The sensitivity of the TMR is symmetric about the *H*_*BFX*_ of about −12 Oe, and it decreases with the increase in the difference between *H*_*BFX*_ with the symmetry line of the *H*_*BFX*_, with an approximate tends to be an inversely proportional function.

### 4.2. TMR Wafer Hysteresis

The TMR device formed by using the two-step annealing process alone has a large hysteresis, which affects the output results of the TMR. According to the results in Section 4.1, the effect of hysteresis cannot be eliminated by changing the annealing parameters and the long-axis lengths of the TMR. Modulating the *H*_*BFX*_ will reduce the hysteresis. Considering the effect of different *H*_*BFX*_ on the hysteresis, the hysteresis optimizing results are obtained as shown in Figure 5i.

Obtain the hysteresis of TMR from the test data and evaluate it using the following methods. TMR hysteresis is only meaningful within a linear range. Linearly fit the TMR resistance output curve of the upward and downward segments separately to obtain the fitting functions of the two branches. Based on this sensitivity, calculate the magnetic field difference between the two branches when they reach the same *R*_*ave*_. This difference value is used as the hysteresis of TMR for evaluation.

The hysteresis of the TMR reaches a level of less than 1 Oe when the *H*_*BFX*_ is more than −40 Oe in the negative direction or more than 17 Oe in the positive direction. The hysteresis curve exhibits similar angular characteristics at different angles of the external field, with a relatively smaller level of hysteresis at 90° external field.

### 4.3. Wheatstone Bridge Structure TMR Sensitivity Results

**Figure 6:**
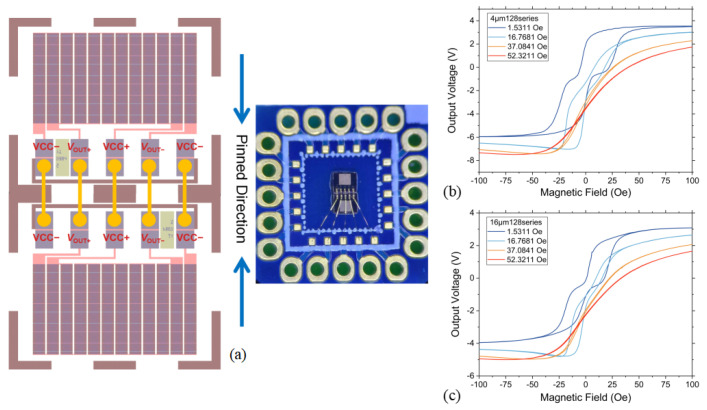
(**a**) Schematic and actual image of the packaged bridge-type TMR; response curve of the Wheatstone bridge structure TMR output with different *H*_*BFX*_. (**b**) Annealed at 350 °C, 6.5 kOe, with a 4 μm long-axis length and 128 series connections, 1 V power supply with a 10-fold amplification gain from the amplifier. (**c**) Annealed at 350 °C, 6.5 kOe, with a 16 μm long-axis length and 128 series connections 1 V power supply with a 10-fold amplification gain from the amplifier.

Furthermore, the TMR devices were configured into a Wheatstone bridge structure, as shown in Figure 1. The packaged device is shown in Figure 6a. Two TMR cells were mounted in opposite orientations on a PCB board, with the sensitive axes of the resistances in the two TMR chips aligned in opposite directions. Gold wires were used to connect the corresponding pins of the TMR, resulting in a packaged bridge-type TMR sensor.

To assess the impact of *H*_*BFX*_ on the TMR output response, a series of magnetic fields bias with varying intensities were applied along the easy axis of the TMR using a biaxial Helmholtz coil. The output response was then precisely measured and recorded as the magnetic field along the hard axis was varied. Testing was conducted on a TMR sensor with 128 series connections and $H_{0}=6.5$ kOe. Typical test results are shown in Figure 6b,c display the output curve corresponding to long-axis lengths of 4 μm and 16 μm, respectively.

After applying $H_{\mathrm{BFX}}$, a significant reduction in hysteresis was observed for the TMR device through Figure 6b,c. Clear linear regions appeared in the output curve for bias fields greater than 37 Oe. The fitted sensitivity of the TMR resistance output curve for the measured magnetic field variations is summarized in Table 2 and Table 3. Table 2 presents the sensitivity fitting results for the magnetic field decreasing process, while Table 3 shows the results for the magnetic field increasing process. At lower bias levels, as illustrated in Figure 6b,c, the presence of substantial hysteresis and discontinuities in the linear region means that the fitted sensitivity may not accurately reflect the TMR’s measurement capability. This discrepancy is evident in the significant difference between the sensitivity fitting results for increasing and decreasing fields. When the sensitivity fitting results for the increasing and decreasing processes differ by less than 1%, hysteresis effects are considered negligible. There exists an ideal linear range, only in where the sensitivity is meaningful. Therefore, we select the maximum sensitivity that differs by less than 1% under the same configuration from two tables as the optimal sensitivity.

For TMR sensors with a long-axis length of 64 μm, a relatively high *H*_*BFX*_ = 52.23 Oe is required to achieve a reasonable linear region, resulting in a sensitivity of only 55.8 V/V/T. As the long-axis length of the TMR sensor decreases, sensitivity improves, and the required *H*_*BFX*_ also decreases. For instance, TMR sensors with long-axis length ranging from 16 μm to 4 μm require *H*_*BFX*_ = 47.24 Oe to meet the 1% sensitivity error criterion, with maximum sensitivity reaching 112.88 V/V/T at a long-axis length of 4 μm.

Analyzing with the *H*_*BFX*_, for the same long-axis-length sensor, increasing the *H*_*BFX*_ reduces the hysteresis and enhances the linearity, but the sensitivity decreases as the *H*_*BFX*_ increases. For the TMR sensors prepared in this paper, the best parameters for sensitivity are 128 series, 4 μm long-axis length, $H_{0}=6.5\;\mathrm{kOe}$, and *H*_*BFX*_=47.24Oe for the TMR sensors.

### 4.4. Measurement of Noise Levels

To evaluate the output noise characteristics of the TMR Wheatstone bridge structure, a systematic data processing procedure was implemented. Specifically, we first applied a Fourier transform to the acquired noise signals, followed by averaging the data 20 times to analyze the spectral characteristics of the noise.

**Figure 7:**
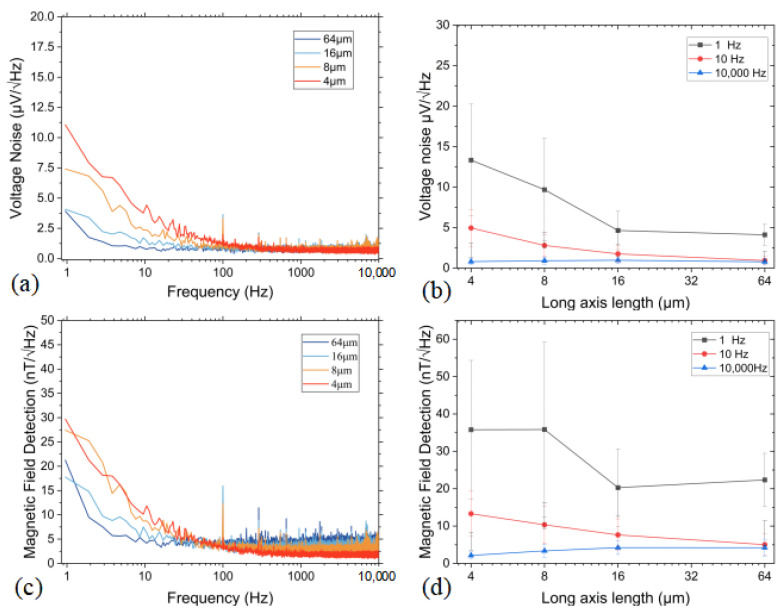
(**a**) Voltage noise curve of the bridge structure. (**b**) Voltage noise with different length of the long axis and its error bars. (**c**) Magnetic field detection of the bridge structure. (**d**) The detection with different long-axis lengths and its error bars.

We selected a TMR sensor with a number of 128 series MTJ connections as a test subject and investigated the effect of the variable of long-axis length on its noise performance. Through precise measurements and analysis, the corresponding results were obtained, as shown in Figure 7a. From the analysis, it is evident that as the long-axis length of the TMR decreases, the low-frequency voltage noise level also decreases. When the long-axis length is reduced to 4 μm, the low-frequency voltage noise level reaches the 10 μV. However, when the long-axis length increases to 16 μm, the low-frequency voltage noise level drops to around 4 μV. Combined with the sensitivity analysis in Section 4.3, the magnetic field noise level was derived, as shown in Figure 7b.

For devices with long-axis lengths of 64 μm and 16 μm, sensitivity was measured under a magnetic field bias of 52.32 Oe, resulting in sensitivities of 55.81 V/V/T and 69.43 V/V/T, respectively. For devices with long-axis lengths of 8 μm and 4 μm, sensitivity was measured under a magnetic field bias of 47.21 Oe, yielding sensitivities of 81.88 V/V/T and 112.88 V/V/T, respectively. Considering that the supply voltage for measuring TMR voltage noise comes from a low-noise 3.3 V battery, it is important to ensure that the equivalent supply voltages are consistent, when calculating the magnetic field noise based on the voltage noise and sensitivity.

At a frequency of 1 Hz, the device with a long-axis length of 16 μm exhibited the best magnetic field detection, with a value of 17.7 nT/V/$\sqrt{\mathrm{Hz}}$@1Hz, as shown in Figure 7c (light blue line). Compared to the other points in Figure 7d, the long-axis lengths of 16 μm and 64 μm exhibit better performance at low frequencies. For noise measurement, the main source of error are white noise and randomness. In the statistics of the error bars, the standard deviation of the noise is approximately between 30% and 50%. This parameter combination produced a TMR sensor with improved magnetic field measurement capabilities, offering the potential for weak magnetic field detection.

## 5. Conclusions

In this research, we fabricated and characterized a series of MTJs with elliptical cross-sections and varying long-axis lengths. We investigated the effects of series connections, long-axis lengths, annealing magnetic fields, and bias magnetic field parameters on TMR sensitivity, as well as the impact of long-axis lengths on noise. The TMR transmission curve demonstrated that TMR sensitivity is closely related to size parameters. Sensors with elliptical free layers and smaller MTJ areas exhibited extremely high sensitivity. Furthermore, increasing the long-axis lengths significantly reduced the voltage noise power in the low-frequency region. Considering the overall detection, TMR devices with a long-axis length of 16 μm achieved superior magnetic field detection capability, which is consistent with the predicted results, reaching a detection level in the 17.7 nT/$\sqrt{\mathrm{Hz}}$@1Hz.

In terms of series connections, TMR sensors with a higher number of series junctions exhibited greater stability and sensitivity. Selecting 128 or more series connections as the TMR configuration parameter allows for the development of highly sensitive sensors.

Excessively high annealing magnetic fields can degrade the crystalline structure, thereby reducing TMR sensitivity, while excessively low annealing fields fail to pin the reference layer in the hard axis direction, also leading to reduced sensitivity. In this research, an annealing magnetic field of 6.5 kOe was selected to optimize TMR sensitivity.

The $H_{\mathrm{BFX}}$ can eliminate the hysteresis effect in TMR; however, a high bias reduces sensitivity. Thus, a bias magnetic field of 47.24 Oe was chosen to maximize sensitivity while keeping hysteresis within a reasonable range.

Experimental results indicate that the long-axis lengths of the TMR plays a crucial role in detecting magnetic field variations, while the number of series junctions and the annealing magnetic field primarily influence the stability of the TMR, with only minor effects on sensitivity. These results provide significant guidance for the optimization of TMR sensors design, particularly in the design of the high-sensitivity and low-noise TMR sensors. 

## Figures and Tables

**Table 1:** Output MR of TMRs with different numbers of series connections.

Number of Series Connections	Length of Long Axis (μm)				
	**64**	**32**	**16**	**8**	**4**
16	13.29	17.47	27.05	46.66	7.54
32	17.47	16.43	36.46	51.40	6.045
64	29.33	44.66	54.88	54.89	64.41
128	43.64	48.27	64.28	66.89	82.98
256 (192)	57.33	58.75	67.68	79.06	87.60
512 (384)	59.46	61.06	68.61	77.88	88.68

**Table 2:** Fitted sensitivity of the downward curve for 128-series TMR.

Sensitivity of TMR (V/V/T)		Length of Long Axis (μm)			
		**64**	**16**	**8**	**4**
*H*_*BFX*_(Oe)	1.5311	79.18	173.66	142.41	212.38
	16.7681	70.05	105.96	110.06	131.71
	37.0841	87.34	100.60	109.05	150.12
	42.1631	80.56	87.70	101.68	142.43
	47.2421	73.73	76.43	81.21	111.96
	52.3211	55.74	69.27	75.02	100.11

**Table 3:** Fitted sensitivity of the upward curve for 128-series TMR.

Sensitivity of TMR (V/V/T)		Length of Long Axis (μm)			
		**64**	**16**	**8**	**4**
*H*_*BFX*_(Oe)	1.5311	746.8	137.92	154.69	285.99
	16.7681	190.85	246.82	274.91	385.61
	37.0841	67.89	85.57	89.10	132.58
	42.1631	64.07	82.30	87.64	127.44
	47.2421	62.68	77.36	81.88	112.88
	52.3211	55.81	69.43	75.31	100.73

## Data Availability

Data are contained within the article.
